# Traditional Uses, Phytochemistry, and Pharmacological Properties of the Genus *Blechnum*—A Narrative Review

**DOI:** 10.3390/ph15070905

**Published:** 2022-07-21

**Authors:** Emmanuel Nyongesa Waswa, Felix Wambua Muema, Wyclif Ochieng Odago, Elizabeth Syowai Mutinda, Consolata Nanjala, Elijah Mbandi Mkala, Sarah Getachew Amenu, Shi-Xiong Ding, Jing Li, Guang-Wan Hu

**Affiliations:** 1 Key Laboratory of Plant Germplasm Enhancement and Specialty Agriculture, Wuhan Botanical Garden, Chinese Academy of Sciences, Wuhan 430074, China; waswa.emmanuell@gmail.com (E.N.W.); fwambua83@mails.ucas.ac.cn (F.W.M.); wyclifodago88@gmail.com (W.O.O.); elizabeth@wbgcas.cn (E.S.M.); nanjala.conso@gmail.com (C.N.); mkala@wbgcas.cn (E.M.M.); endale.tamiru@mails.ucas.ac.cn (S.G.A.); shixiongdingjx@163.com (S.-X.D.); lijing204@mails.ucas.ac.cn (J.L.); 2 Center of Conservation Biology, Department of Botany, Core Botanical Gardens/Wuhan Botanical Garden, Chinese Academy of Sciences, Wuhan 430074, China; 3 Sino-Africa Joint Research Center, Chinese Academy of Sciences, Wuhan 430074, China; 4 University of Chinese Academy of Sciences, Beijing 100049, China

**Keywords:** *Blechnum*, pharmacology, phytochemistry, traditional uses

## Abstract

*Blechnum* L. is a genus belonging to the Blechnaceae family with 236 accepted species that grow in intertropical, subtropical, and southern temperate regions. Several species of the genus have long been used in folk medicines to treat a broad spectrum of ailments, including typhoid, urinary infections, influenza, wounds, pulmonary complaints, blisters, boils, and antihelmintic-related complications. So far, about 91 chemical compounds have been isolated from different parts of 20 *Blechnum* species. Among these metabolites, phenolic compounds, sterols, and fatty acids are the main constituents. Modern pharmacological investigations revealed several isolated compounds and extracts to exhibit exceptional biological properties including the antioxidant, antimicrobial, anti-inflammatory, anticancer, insecticidal, antitrematocidal and wound healing. In various tests, both quercetin-7′,3′,4′-trimethoxy and phytol metabolites showed potential antioxidant and antitrematocidal properties, while ponasterone exhibited insecticidal activity. Despite having a broad range of traditional medicinal benefits and biological properties, understanding the scientific connotations based on the available data is still challenging. This article presents a comprehensive review of the traditional uses, phytochemical compounds, and pharmacological aspects of the *Blechnum* species.

## 1. Introduction

Ancient traditional knowledge of plants’ medicinal utility contributes significantly to bioprospecting by identifying plants for drug discovery. Many medicinal plants are effective remedies for diverse complaints, prompting increased screening for their therapeutic constituents [1,2]. In the past, pteridophytes (fern and fern-allies) were considered as significant sources of treatment against different disorders and for culinary purposes [3,4,5].

*Blechnum* L. (Blechnaceae) is a fern genus with approximately 236 species (https://powo.science.kew.org/, accessed on 7 April 2022) [6]. They are widespread in tropical regions [7] and encompass terrestrial, rupestral, climbing, or epiphytic plants. The species of this genus are referred to as hard or midsorus ferns. Several *Blechnum* species are used as herbal medicines to cure sicknesses such as pulmonary complaints, typhoid, influenza, urinary bladder, intestinal worms, wounds, blisters, and boils [1,8,9].

Natural agents with medicinal values have been extensively investigated in plants [10], and various studies have explored the phytocompounds and bioactivities of different *Blechnum* species. *Blechnum* is a novel source of alcohols, aldehydes, carotenoids, phenolic acids, sesquiterpene [11], fatty acids [12], phytosterols [13], steroids [14], lignans [15], flavonols, flavones [2,16], anthocyanidins [17], and diterpenes [18]. *Blechnum* species have been examined for pharmacological properties such as antioxidant [12], antimicrobial [19], anticancer [20], insecticidal [21], wound healing [22], antitrematocidal [23], and anti-inflammation [5]. A comprehensive description of the biological activities exhibited by the *Blechnum* species is expounded in the pharmacology section.

Even though several *Blechnum* species are globally employed in folk medicines, secondary metabolites and their biological activities have not been extensively investigated. Thus, the scientific basis for their traditional medicinal applications is poorly understood. The present article discusses the traditional uses, phytochemistry, and biological activities of the *Blechnum* species.

In this review, the scientific data was retrieved from published articles using various electronic search engines like PubMed, Web of Science, and Google Scholar. The searchers were not restricted to the quality of publication or language used. The Global Biodiversity Information Facility (GBIF) (https://www.gbif.org/, accessed on 7 April 2022), Flora of China (http://www.efloras.org, accessed on 7 May 2022), Plants of the World Online (POWO) (https://powo.science.kew.org/, accessed on 7 April 2022), and the International Union for Conservation of Nature (IUCN) (https://www.iucnredlist.org/, accessed on 18 April 2022) were used to gather information on the botanical description, distribution, and conservation status of *Blechnum* plants. The keywords used include *Blechnum*, distribution, physical characteristics, conservation, phytochemical compounds, biological activities, and traditional medicinal uses. The scientific names were confirmed using the World Flora online and Plants of the World Online (POWO) (https://powo.science.kew.org/, accessed on 4 May 2022) databases, while phytochemical structures were drawn using ChemBioDraw Ultra v14.0 software. Ninety-six articles were included in this study.

### 1.1. Botanical and Taxonomic Description

**Figure 1:**
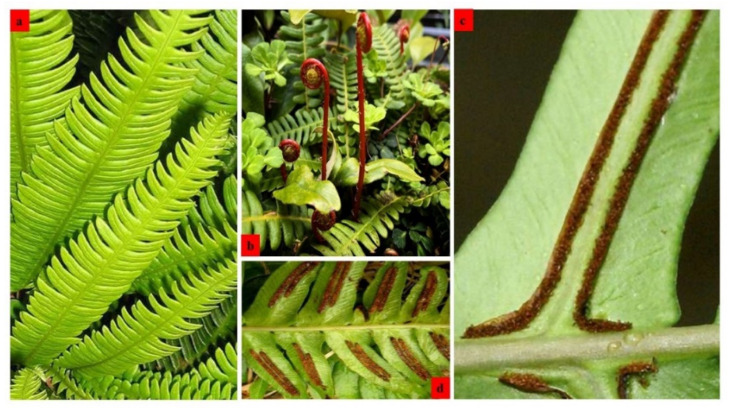
(**a**) Mid-green glossy fronds of *Blechnum*, (**b**) Oblong leaflets, (**c**) Leaves with sporangia at the adaxial surface, (**d**) Pinnate sterile/fertile fronds.

*Blechnum* species are distinguished by erect rhizomes which occasionally creep/form short trunks. The rhizomes consist of unarticulated stipes covered with protective hairlike scales, and in most *Blechnum,* they tend to exist primarily as underground structures. Leathery fronds are monomorphic or dimorphic [7], while circinate venation, simple lamina, and sometimes 1-pinnatifid or 1-pinnate characterize their stalks and leaf blades (Figure 1). The sterile and fertile fronds can either be pinnate or bipinnate (http://www.worldfloraonline.org, accessed on 11 April 2022). Rachis and costae are scaly and glabrous, whereas sori are paralleled next to costae and are borne on vascular commissures. They reproduce with spores as substitutes for seeds produced in bundles under the fronds. The spores are perine smooth to diversely winged or rugose, with reticulate perispore, and contain small papillae [7].

The genus *Blechnum* is found in the Blechnaceae family, subfamily Blechnoideae, and order Polypodiales. It is a taxonomically complex paraphyletic genus with unclear internal relationships. As presently conceived, this genus encompasses the variously formerly segregated genera including *Spicanta* C. Presl, *Lomaridium* C. Presl, *Lomaria* Willd., *Blechnopsis* C. Presl, *Struthiopteris* Scop., and *Distaxia* C. Presl [24]. It comprises approximately 236 globally accepted species and 29 synonyms (https://powo.science.kew.org/, accessed on 11 April 2022). In addition, two accepted subspecies and three varieties were described (http://www.worldfloraonline.org, accessed on 11 April 2022).

### 1.2. Distribution and Conservation Status

**Figure 2:**
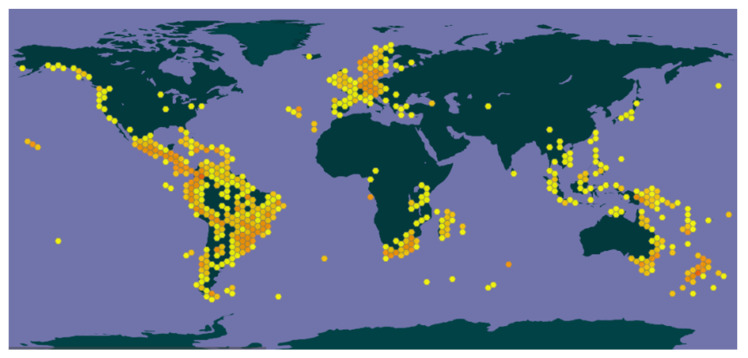
Distribution of *Blechnum* species (GBIF).

*Blechnum* species are broadly distributed in various parts of America, Europe, Africa, Australia, Asia, and several islands, including Hawaii, Mascarene, and Cunha among others (https://powo.science.kew.org/, accessed on 7 April 2022) [25] (Figure 2). They mainly occupy terrestrial, rocky areas and can exist as erect, climbing, or rarely epiphytic plants. In New Zealand, they are represented by about 18 indigenous species. In China, the *Blechnum orientale* (L.) C. Presl., is widespread in Fujian, Guangdong, Hainan, Hunan, Sichuan, Yunnan, Zhejiang, and neighboring areas (http://www.efloras.org, accessed on 7 April 2022). Additionally, 15 primarily epiphytic and terrestrial *Blechnum* plants are widespread in Mexico [7], whereas 13 are common in Chile [26]. Most of these species are sub-cosmopolitan and inhabit the intertropical, subtropical, and southern temperate regions. The conservation status of about 145 recognized *Blechnum* species are yet to be evaluated (https://www.iucnredlist.org/, accessed on 18 April 2022). Several species, including *Blechnum attenuatum* (Sw.) Mett., *B. punctulatum* Sw., *B. australe* L., and *B. heringeri* Brade. are listed as Least Concern (LC). In addition, *B. eburneum* Christ., *B. monomorphum* R.C. Moran & B. Øllg, and *B. occidentale* L., *(B. sodiroi* C. Chr) are reported as Vulnerable (VU), whereas *B. floresii* (Sodiro) C. Chr., is Endangered (EN).

## 2. Traditional Uses of *Blechnum* Species

**Figure 3:**
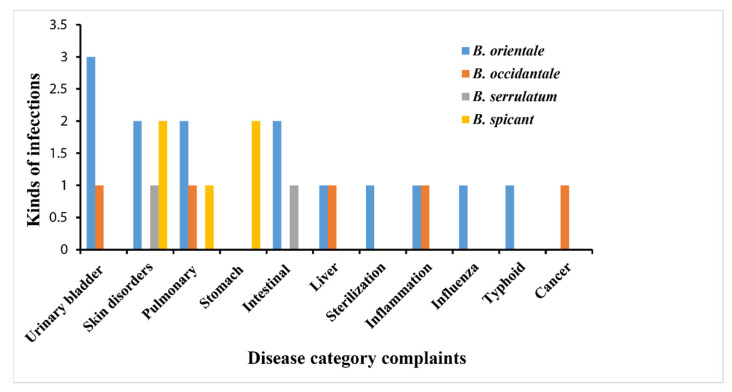
Traditional herbal uses of *Blechnum* species.

*Blechnum* species are used in Chilean Traditional Medicines (CTM), Traditional Indian Medicines (TIMs), and Traditional Chinese Medicines (TCMs) for various medicinal folklore [26,27,28]. The different types of *Blechnum*-derived formulations in the form of decoction, poultice, infusion, paste, and juice (Table 1) prepared from the fronds, rhizomes, whole plants, roots, and shoots are remedies for cancer, typhoid, influenza, inflammation, female sterilization, liver infections, intestinal wounds, stomach complaints, pulmonary illnesses, skin disorders, and urinary bladder complaints (Figure 3, Table 1)*. Blechnum occidentale* L., *B. spicant* (L.) Roth, *B. serrulatum* Rich, and *B. orientale* L. were found to be the most commonly utilized *Blechnum* species for medicinal purposes (Table 1).

**Figure 4:**
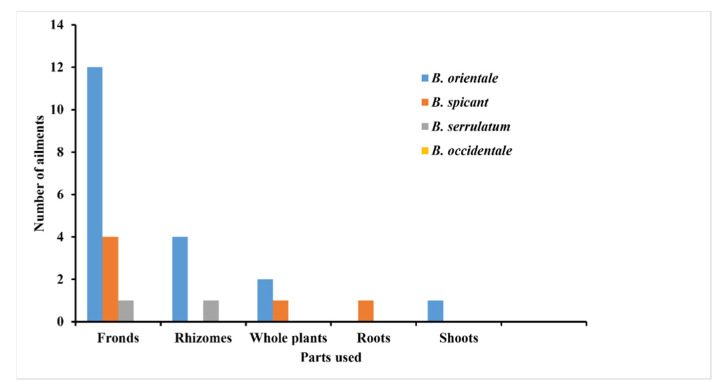
Application of *Blechnum* species against different ailments.

Utilization of plants for medicinal purposes is attributed by their rich active ingredients [29]. They are utilized for clinical drug development as alternative natural agents to conventional chemotherapies. Traditional knowledge indicates that different parts of the *Blechnum* plants (Figure 4) possess medicinal efficacy towards diverse sicknesses, thereby contributing to human health. *Blechnum orientale* was established as the most commonly used species to prepare folk medicines in the studied genus. The frequently used parts in the preparation of herbal medicines included fronds and rhizomes, while roots and shoots are less widely used (Figure 4).

The fronds obtained from *B. orientale* are regarded as cure for intestinal wounds, pulmonary complaints, skin disorders, and urinary bladder infections. Additionally, those obtained from *B. spicant* are chewed to cure skin disorders, cancer, pulmonary, and stomach complaints, while *B. serrulatum* is used as a remedy for skin disorders. The rhizomes from *B. orientale* and *B. serrulatum* are also significant sources of traditional therapies for urinary bladder infections, typhoid, and intestinal worms (Table 1). Decoction prepared from the roots of *B. spicant* is used to cure diarrhoea, while the shoots obtained from *B. orientale* are pounded and the paste is applied to treat boils [30,31]. Oral ingestion of the entire plant of *B. orientale* has a medicinal relevance in the sterilization of women. Likewise, the whole plants of *B. spicant* are popular medicines against skin ailments in the United States of America [32].

**Table 1:** Traditional medicinal uses of the *Blechnum* species.

Accepted Species	Region	Parts Used	Medicinal Use	References
*B. spicant* (L.) Roth.		Fronds	The leaflets are chewed to treat internal cancer, lungs, and stomach complaints.	[30]
			They are externally applied to treat skin sores.	[30]
		Roots	Decoctions prepared from the roots are used as a remedy for diarrhea	[30]
	United States of America	Whole plant	Treatment of skin ailments	[32]
*B. orientale* (L.) C. Presl.	Malaysia.	Shoots	The shoots are pounded and used as a paste to cure boils	[31]
		Fronds	The fronds are ground in cow’s milk to treat asthma	[1]
			Applied in the form of a poultice to treat boils	[31]
			Externally applied to cure blisters, boils, carbuncles, and sores	
			The leaves are crushed and applied as medication for abscesses	[31,32,33]
	India	Fronds	Urinary bladder complaints	[8,34]
			Hot decoction prepared from pinnae is used for its antiseptic action or applied externally over a boil to release pus.	[35]
			The extracted juice is used to treat intestinal Wounds.	[36]
		Rhizomes	It is orally administered to treat typhoid.	[9,32]
			The prepared paste is applied to cure urinary bladder infections.	[36]
	Philippines	Fronds	They are used for polynesia, diaphoretic, and operative actions	[8,28]
	China	Rhizomes	Are used as an anthelminthic to cure intestinal worms	[8,28]
	Papua New Guinea	Whole plant	Orally ingested for women’s sterilization	[32]
	United States of America		Treatment of influenza	[37]
*B. occidentale* L.	Brazil	-	The whole plant is frequently used as a therapy for pulmonary ailments, urinary disorders and liver infections.	[3,38,39]
	-	-	Inflammation	[40]
*B. serrulatum* Rich.	French Guiana	Rhizome	An infusion prepared from the rhizomes is used as a vermifuge.	[41]
	Guyana	Fronds	Used to treat abscesses	[41]

## 3. Secondary Metabolites

**Figure 5:**
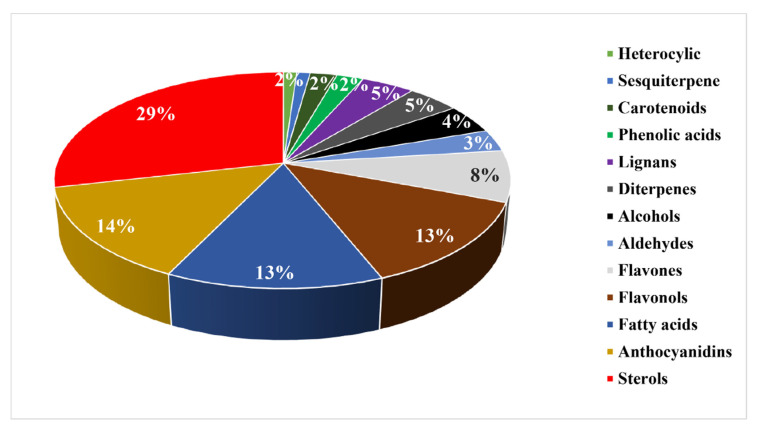
Chemical compounds isolated from *Blechnum* species.

Early phytochemical study of the *Blechnum* genus occurred in the 1960s, which revealed the existence of chlorogenic and blechnic acid in the leaves of *B. spicant, B. orientale, B. discolor*, and *B.* *brasiliense* [42]. Later, preliminary phytochemical studies identified numerous anthocyanidins from the leaves of *B. procerum* [17]. Other investigations in subsequent years resulted in isolation and identification of steroids, flavonols, flavones, lignans, fatty acids, phytosterols, diterpenes, sesquiterpenes, alcohols, aldehydes, carotenoids, and heterocyclic compounds (Figure 5).

In the present study, a detailed investigation of phytochemicals derived from the *Blechnum* species revealed a broad spectrum of structurally diverse and biologically active metabolites. Ninety-one specialized metabolites encompassing 39 phenolic compounds, 25 sterols, 12 fatty acids, 5 terpenes, 4 alcohols, 3 aldehydes, 2 carotenoids, and a heterocyclic compound were isolated (Table 2). The dominant specialized products were steroids, anthocyanidins, flavonoids, and fatty acids, whereas carotenoids, sesquiterpenes, and heterocyclic metabolites were the least isolated. Chemical compounds were primarily screened from leaves/fronds, rhizomes, whole plants, and aerial parts and are described based on the original sources. The major techniques used screen metabolites included Liquid Chromatography, Mass spectrometry (MS) and Nuclear Magnetic Resonance (NMR).

### 3.1. Phenolic Compounds

**Figure 6:**
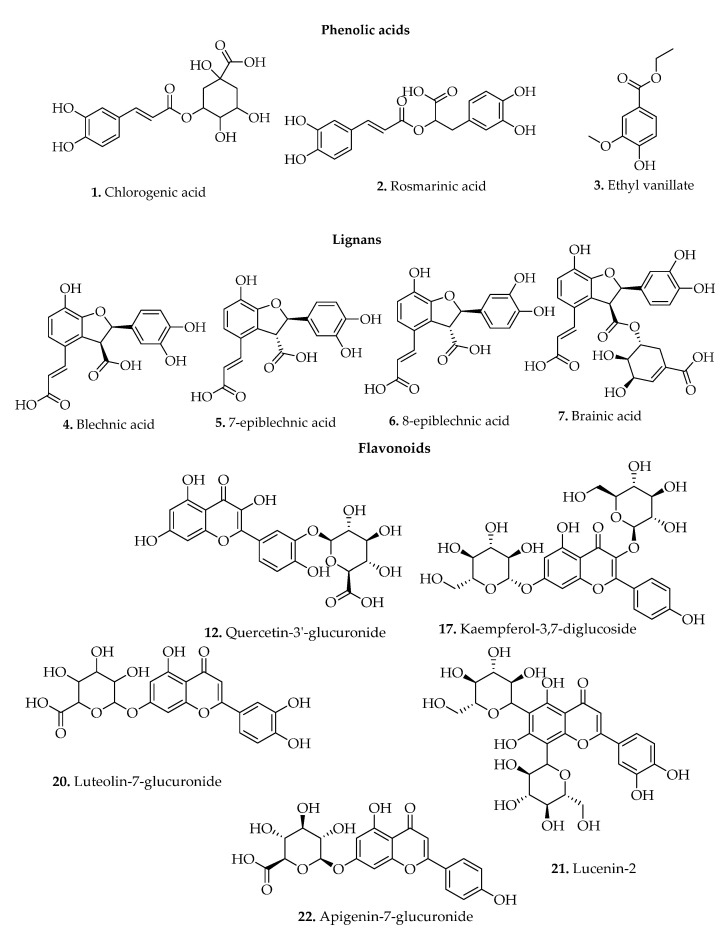
Chemical structures of some phenolic compounds isolated from *Blechnum* species.

Phenolic compounds are among plants main classes of natural bioactive metabolites and are structurally distinguished by a single phenol unit, broadly grouped as simple phenols and polyphenols [43]. These metabolites were isolated from several *Blechnum* species, and 40 secondary metabolites are reported (Table 2), with some of their chemical structures depicted in Figure 6.

#### 3.1.1. Simple Phenols/Phenol Derivatives

Numerous *Blechnum* species were reported as essential sources of rosmarinic and chlorogenic acid constituents [42,44,45]. The above-investigated compounds were found in the extracts prepared from the leaves of *B. binervatum, B. brasiliense, B. orientale, B. discolor, B. brasiliense, B. spicant, and B. occidentale* (compounds **1** and **2**) (Table 2). Ethyl vanillate (**3**) metabolite was also detected from the aerial parts of *B. spicant* [11]. Rosmarinic acid (RA) screened from various medicinal plants has interesting health-promoting effects including antioxidant, anti-inflammatory, and antimicrobial properties [46,47,48]. In addition, numerous studies indicate that chlorogenic acid exhibits promising pharmacological properties [49,50,51].

#### 3.1.2. Lignans

Lignans phytochemicals have been highly screened in various Blechnaceae species [52]. In the *Blechnum* genus, these compounds (**4**–**7**) were isolated from the fronds of *B. orientale* and *B. spicant* (Table 2). The investigation by Wada et al. [15] assessed *B. orientale* extracts and detected various metabolites including blechnic acid, 7-epiblechnic acid, 8-epiblechnic acid, and brainic acid. Figure 6 shows the chemical structures of some of the isolated lignans.

#### 3.1.3. Polyphenols

##### Flavonols

Flavonoid compounds, a broad group of polyphenols with the C15-carbon skeleton, are highly distributed in plants and are linked with extensive health benefits such as antimicrobial, antiparasitic, anti-inflammation, and anticancer [53]. Several flavonol metabolites, primarily quercetin derivatives (**8**–**15**) and kaempferol derivatives (**16**–**19**), were detected from the leaves and aerial parts of *B. novae-zelandiae* and *B. orientale* (Table 2).

##### Flavones

Flavones are structurally comprised of 2-phenyl-1,4-benzopyrone backbones, and their occurrence has been investigated in the studied genus. Several studies have reported flavones in *B. orientale* extracts [2,16,23]. Seven flavones (**20**–**26**) constituting the luteolin (luteolin-7-glucuronide (**20**) and lucenin-2 (**21**)) and apigenin derivatives (**22**–**26**) were screened and identified from the aerial parts of *B. orientale* (Table 2).

##### Anthocyanins

Anthocyanins are naturally-pigmented, water-soluble, bioactive flavonoid compounds [54]. The sugar-free analogues of anthocyanins are anthocyanidins. Approximately seven anthocyanidins metabolites were detected from the extracts of *B. procerum* and *B. novae-zelandiae* (**27**–**39**) [17,55]. Anthocyanidins are brightly coloured anthocyanin-like pigments distinguished from C-3 oxygenated flavonoids by two doublets of heterocycle at the H-3 and H-4 positions. The 3-deocyanthocyanins metabolites consisting of apigeninidin (**27**–**32**) and luteolinidin (**33**–**39**) derivative groups were screened from the fronds of *B. procerum* and *B. novae-zelandiae* (Table 2).

### 3.2. Terpenoids

**Figure 7:**
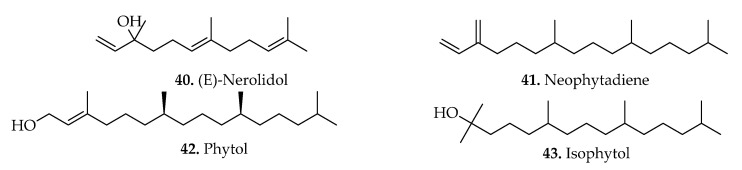
Chemical structures of terpenoids isolated from *Blechnum* species.

Terpenic compounds are amongst the widely isolated groups of phytochemicals in the Blechnaceae family [23,26]. The number of known screened terpenes in the *Blechnum* genus is relatively high compared to other specialized metabolites. Approximately five terpenes (compounds **40**–**44**) were isolated (Table 2), and some of their chemical structures are illustrated (Figure 7). The aerial parts of *B. spicant* contain (*E*)-nerolidol sesquiterpene (compound **40**) [11]. This metabolite possesses several biological activities, including anticancer, antioxidant, and antimicrobial [56,57,58] effects. Furthermore, several diterpenes (**41**–**44**) were isolated from the leaves and aerial parts of various *Blechnum* plants.

### 3.3. Sterols

**Figure 8:**
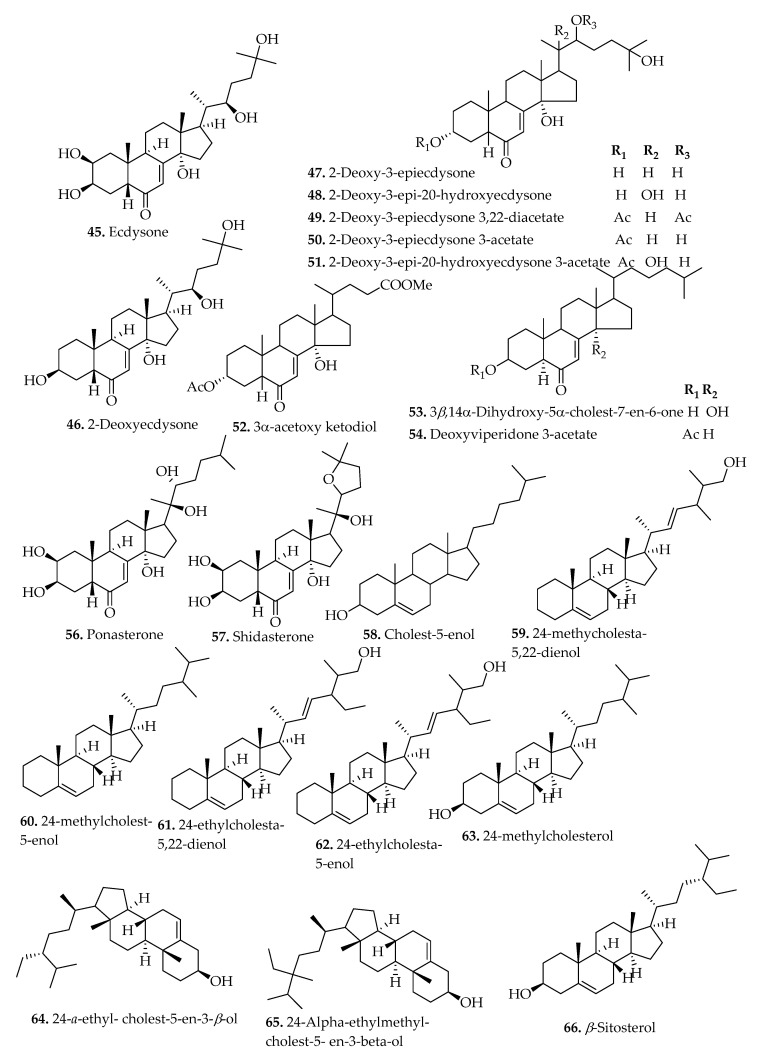
Chemical structures of sterols isolated from *Blechnum* species.

Sterol metabolites have been highly isolated and identified in the family Blechnaceae [12,13,18]. They are isoprenoid derivatives with diverse composition existing as either free forms, conjugated, or steryl glycosides [59]. Sterol compounds with high structural diversity were isolated from numerous *Blechnum* plants (compounds **45**–**69**) (Table 2), and some of their structural diversity is represented in Figure 8. The phytoecdysteroids (compounds **45**–**56**): fundamental analogues in insects as moulting hormones were isolated from the fronds of *B. vulcanicum* [14]. Similarly, several other constituents including ecdysone (**45**), ponasterone (**56**), and shidasterone (**57**), were also screened from dried leaves of different *Blechnum* species. Compound (**56**) and its synthesized derivatives exhibited receptor-binding activities towards the ecdysone receptor of Kc cells [60].

The phytosterol compounds were also isolated from the whole plant extracts of *B. orientale* [12,13,18]. These are plant-derived fatty compounds with limited structural diversity and are cholesterol-related [10]. Numerous previous investigations reported the biological properties of the phytosterols, and nine compounds (**66**–**69**) were isolated and identified primarily from the extracts of *B. corralense*, *B. chilense*, *B. brasiliense*, *B. microphyllum. asperum*, *B. binervatum*, *B. mochaenum. blechnoides*, *B. hastatum*, and *B. penna-marina*, *B. magellanicum*, *B. arcuatum*, and *B. occidentale* (Table 2).

### 3.4. Fatty Acids

**Figure 9:**
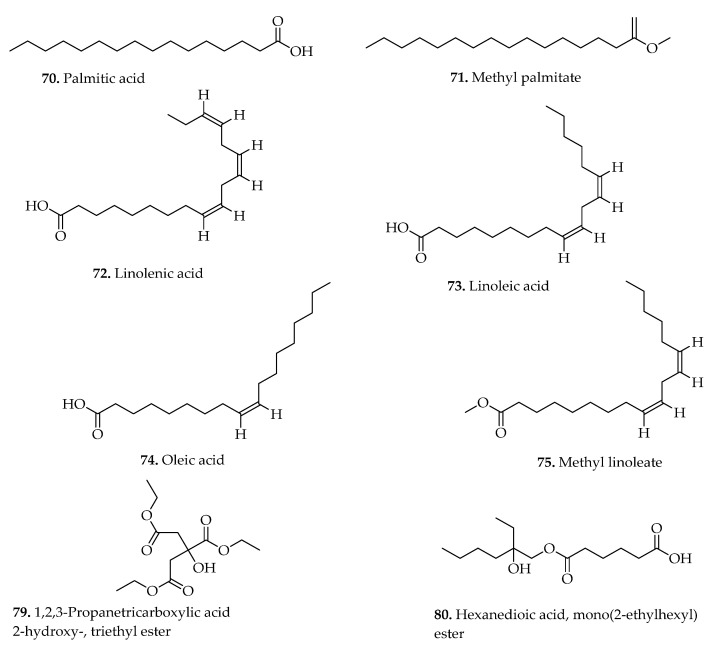
Representative chemical structures of fatty acids isolated from *Blechnum* species.

Fatty acids encompass saturated and unsaturated hydrocarbon chains of variable sizes, distinguished by the carboxyl group positioned at one end, and the methyl group at the inverse [61]. Numerous studies have reported several fatty acids from plants to be linked to various biological properties [62,63]. In the present investigation, 12 structurally diverse fatty acid constituents (compounds **70**–**81**) were isolated from the leaves and whole plants of different *Blechnum* plants [11,12,23], and their representative chemical structures shown (Figure 9, Table 2). The phytochemical analysis by Maria et al. [12] revealed several fatty acid components (**70**–**78**) from dichloromethane and hexane fractions prepared from the leaves of *B. binervatum*, *B. occidentale*, and *B. brasiliense*. Similarly, two fatty acid components; 1,2,3-propanetricarboxylic acid 2-hydroxy-, triethyl ester (**79**), and hexanedioic acid, mono (2-ethylhexyl) ester (**80**) were isolated from *B. orientale* extracts and registered to exhibit numerous medicinal properties including antimicrobial, anti-inflammatory, antioxidant, antiulcer, and nematicidal [23].

**Table 2:** Screened secondary metabolites from the *Blechnum* species.

No.	Secondary Metabolite	Specimen	Part Used	Identification Method	References
	**Phenolic compounds**				
	**(a). Phenolic acids**				
1.	Chlorogenic acid	*B. binervatum* (Poir.) C.V.Morton & Lellinger, *B. brasiliense* Desv, *B. orientale* L., *B. discolor* (Forst.) Keyserl, *B. brasiliense* Desv., *B. spicant* (L.) Roth, and *B. occidentale* L.	Fronds	TLC, HPLC-DAD-MS	[42,44]
2.	Rosmarinic acid	*B. binervatum* (Poir.) C.V.Morton & Lellinger, *B. brasiliense* Desv., and *B. occidentale* L.	Fronds	TLC, HPLC-DAD-MS	[42,44,45]
3.	Ethyl vanillate	*B. spicant* (L.) Roth	Aerial parts	GC-MS	[11]
	**(b). Lignans**				
4.	Blechnic acid	*B. spicant* (L.) Roth, and *B. orientale* L.	Fronds	TLC	[15,42]
5.	7-Epiblechnic acid	*B. orientale* L.	Fronds		[15]
6.	8-Epiblechnic acid	*B. orientale* L.	Fronds		[15]
7.	Brainic acid	*B. orientale* L.	Fronds		[15]
	**(c). Flavonols**				
8.	Quercetin-3,7-digalactoside	*B. orientale* L.	Fronds	TLC	[2,16]
9.	Quercetin-7,4′-digalactoside	*B. orientale* L.	Fronds	TLC	[2,16]
10.	Quercetin-3,4′-diglucoside	*B. orientale* L.	Fronds	TLC	[2,16]
11.	Quercetin-3′,4′di methyl ether-3-glucoside	*B. orientale* L.	Fronds	TLC	[2,16]
12.	Quercetin-3, glucuronide	*B. orientale* L.	Fronds	TLC	[2,16]
13.	Quercetin 7′3′4′—Trimethoxy	*B. orientale* L.	Aerial parts	GC-MS	[23]
14.	Quercetin 3-*0*-*β-D*-[6-0-caffeoylglucopyranoside]	*B. novae-zelandiae* T.C.Chambers & P.A.Farrant	Fronds	HPLC, NMR	[64]
15.	Quercetin 3-*0*-*β-D*-[6-0-caffeoylgalactopyranoside]	*B. novae-zelandiae* T.C.Chambers & P.A.Farrant	Fronds	HPLC, NMR	[64]
16.	Kaempferol-3,7-digalactoside	*B. orientale* L.	Fronds	TLC	[2,16]
17.	Kaempferol-3,7-diglucoside	*B. orientale* L.	Fronds	TLC	[2,16]
18.	Kaempferol-3,7-diglucuronide	*B. orientale* L.	Fronds	TLC	[2,16]
19.	Kaempferol 3-*0*-*β-D*-glucuronopyranoside	*B. novae-zelandiae* T.C.Chambers & P.A.Farrant	Fronds		[64]
	**(d). Flavones**				
20.	Luteolin-7-glucuronide	*B. orientale* L.	Fronds	TLC	[2,16]
21.	Lucenin-2 (luteolin 6,8-di-C-glucoside)	*B. orientale* L.	Aerial parts		[23]
22.	Apigenin-7-glucuronide	*B. orientale* L.	Fronds	TLC	[2,16]
23.	Isorhamnetin-3-glucoside	*B. orientale* L.	Fronds	TLC	[2,16]
24.	Apigenin-7,4′-diglucoside	*B. orientale* L.	Fronds	TLC	[2,16]
25.	Genkwanin-4′-glucuronide	*B. orientale* L.	Fronds	TLC	[2,16]
26.	Acacetin-7-galactoside	*B. orientale* L.	Fronds	TLC	[2,16]
	**(e). Anthocyanidins**				
27.	Apigeninidin-5-glucoside	*B. procerum* (G.Forst.) Sw	Fronds		[17]
28.	Apigeninidin-7-glucoside	*B. procerum* (G.Forst.) Sw	Fronds		[17]
29.	Apigeninidin-5-diglycoside	*B. procerum* (G.Forst.) Sw	Fronds		[17]
30.	Apigeninidin-7-diglycoside	*B. procerum* (G.Forst.) Sw	Fronds		[17]
31.	Apigeninidin-5-rhamnoside glucoside	*B. procerum* (G.Forst.) Sw	Fronds		[17]
32.	Apigeninidin-5-7-glycoside	*B. procerum* (G.Forst.) Sw	Fronds		[17]
33.	Luteolinidin-5-glucoside	*B. procerum* (G.Forst.) Sw	Fronds		[17]
34.	Luteolinidin-7-glucoside	*B. procerum* (G.Forst.) Sw	Fronds		[17]
35.	Luteolinidin-5-diglycoside	*B. procerum* (G.Forst.) Sw	Fronds		[17]
36.	Luteolinidin-7-diglycoside	*B. procerum* (G.Forst.) Sw	Fronds		[17]
37.	Luteolinidin -5-rhamnoside glucoside	*B. procerum* (G.Forst.) Sw	Fronds		[17]
38.	Luteolinidin-5-7-glycoside	*B. procerum* (G.Forst.) Sw	Fronds		[17]
39.	Luteolinidin 5-*0*-*β*-*D*-[3-0- *ß*-*D*-glucopyranosyl-2-*O*-acetylglucopyranoside]	*B. novae-zelandiae* T.C.Chambers & P.A.Farrant	Fronds	HPLC, NMR	[64]
	**Terpenoids**				
	**(a). Sesquiterpene**				
40.	(*E*)-Nerolidol	*B. spicant* (L.) Roth	Aerial parts	GC-MS	[11]
	**Diterpenes**				
41.	Neophytadiene	*B. penna-marina* (Maxon & C.V.Morton) Kuhn, *B. arcuatum* Remy, *B. mochaenum* G.Kunkel, *B. asperum* (Klotzsch) J.W.Sturm, *B. blechnoides* (Lag.) C.Chr., *B. hastatum* Kaulf, *B. microphyllum* (Goldm.) C.V.Morton, *B. chilense* (Kaulf.) Mett., *B. magellanicum*, *B. corralense* Espinosa, and *B. occidentale* L.	Fronds	GC-MS	[12,18]
42.	Phytol ((3,7,11,15-tetramethyl-2-hexadecen-1-ol)	*B. penna-marina* (Maxon & C.V.Morton) Kuhn, *B. arcuatum* Remy, *B. mochaenum* G.Kunkel, *B. asperum* (Klotzsch) J.W.Sturm, *B. blechnoides* (Lag.) C.Chr., *B. hastatum* Kaulf, *B. microphyllum* (Goldm.) C.V.Morton, *B. chilense* (Kaulf.) Mett., *B. magellanicum* (Desv.) Mett., and *B. corralense* Espinosa.	Fronds	GC-MS	[18]
43.	Isophytol ((3,7,11,15-tetramethyl-1-hexadecen-3-ol)	*B. penna-marina* (Maxon & C.V.Morton) Kuhn, *B. arcuatum* Remy, *B. mochaenum* G.Kunkel, *B. asperum* (Klotzsch) J.W.Sturm, *B. blechnoides* (Lag.) C.Chr., *B. hastatum* Kaulf, *B. microphyllum* (Goldm.) C.V.Morton, *B. chilense* (Kaulf.) Mett., *B. magellanicum* (Desv.) Mett., *B. corralense* Espinosa, and *B. occidentale* L.	Fronds	GC-MS	[12,18]
44.	2-Hexadecene, 3,7,11,15-tetramethyl-, [R-[R*, R*-(E)]]-	*B. orientale* L.	Aerial parts	GC-MS	[23]
	**Sterols**				
45.	Ecdysone	*B. penna-marina* (Maxon & C.V.Morton) Kuhn, *B. arcuatum* Remy, *B. mochaenum* G.Kunkel, *B. asperum* (Klotzsch) J.W.Sturm, *B. blechnoides* (Lag.) C.Chr., *B. hastatum* Kaulf, *B. microphyllum* (Goldm.) C.V.Morton, *B. chilense* (Kaulf.) Mett., *B. magellanicum* (Desv.) Mett., *B. corralense* Espinosa, *B. vulcanicum* (Blume) Kuhn, and *B. minus* (R.Br.) Ettingsh	Fronds	HPLC, GC-MS, NMR	[14,18,26]
46.	2-Deoxyecdysone (2-Deoxycrusteecdysone)	*B. vulcanicum* (Blume) Kuhn, *B. minus* (R.Br.) Ettingsh, *B. arcuatum* Remy, *B. asperum* (Klotzsch) J.W.Sturm, *B. blechnoides* (Lag.) C.Chr., *B. chilense* (Kaulf.) Mett., *B. corralense* Espinosa, *B. hastatum* Kaulf, *B. magellanicum* (Desv.) Mett., *B. microphyllum* (Goldm.) C.V.Morton, *B. mochaenum* G.Kunkel, and *B. penna-marina* (Maxon & C.V.Morton) Kuhn	Fronds	HPLC, GC-MS, NMR	[14,18,26]
47.	2-Deoxy-3-epiecdysone	*B. vulcanicum* (Blume) Kuhn	Fronds	HPLC	[14]
48.	2-Deoxy-3-epi-20-hydroxyecdysone	*B. vulcanicum* (Blume) Kuhn	Fronds	HPLC	[14]
49.	2-Deoxy-3-epiecdysone 3,22-diacetate	*B. vulcanicum* (Blume) Kuhn	Fronds	HPLC	[14]
50.	2-Deoxy-3-epiecdysone 3-acetate	*B. vulcanicum* (Blume) Kuhn	Fronds	HPLC	[14]
51.	2-Deoxy-3-epi-20-hydroxyecdysone 3-acetate	*B. vulcanicum* (Blume) Kuhn	Fronds	HPLC	[14]
52.	3α-Acetoxy ketodiol	*B. vulcanicum* (Blume) Kuhn	Fronds		[14]
53.	3*β*,14α-Dihydroxy-5α-cholest-7-en-6-one	*B. vulcanicum* (Blume) Kuhn	Fronds	HPLC	[14]
54.	Deoxyviperidone 3-acetate.	*B. vulcanicum* (Blume) Kuhn	Fronds	HPLC	[14]
55.	3*β*,14α-Dihydroxy-5β-cholest-7-en-6-one	*B. vulcanicum* (Blume) Kuhn	Fronds	HPLC	[14]
56.	Ponasterone	*B. penna-marina* (Maxon & C.V.Morton) Kuhn, *B. arcuatum* Remy, *B. mochaenum* G.Kunkel, and *B. asperum* (Klotzsch) J.W.Sturm. *B. blechnoides* (Lag.) C.Chr., *B. hastatum* Kaulf, *B. microphyllum* (Goldm.) C.V.Morton, *B. chilense* (Kaulf.) Mett., *B. magellanicum* (Desv.) Mett., and *B. corralense* Espinosa.	Fronds	GC-MS, NMR	[18,26]
57.	Shidasterone	*B. penna-marina* (Maxon & C.V.Morton) Kuhn, *B. arcuatum* Remy, *B. mochaenum* G.Kunkel, *B. asperum* (Klotzsch) J.W.Sturm, *B. blechnoides* (Lag.) C.Chr., *B. hastatum* Kaulf, *B. microphyllum* (Goldm.) C.V.Morton, *B. chilense* (Kaulf.) Mett., *B. magellanicum* (Desv.) Mett., and *B. corralense* Espinosa.	Fronds	GC-MS, NMR	[18,26]
58.	Cholest-5-enol	*B. orientale* L.	Whole plant	GC	[13]
59.	24-methycholesta-5,22-dienol	*B. orientale* L.	Whole plant	GC	[13]
60.	24-methylcholest-5-enol	*B. orientale* L.	Whole plant	GC	[13]
61.	24,-ethylcholesta-5,22-dienol	*B. orientale* L.	Whole plant	GC	[13]
62.	24-ethylcholest-5-enol	*B. orientale* L.	Whole plant	GC	[13]
63.	24-methylcholesterol	*B. orientale* L.	Whole plant	GC	[13]
64.	24-α-ethyl-cholest-5-en-3β-ol	*B. orientale* L.	Whole plant	GC	[13]
65.	24-Alphaethyl-methyl-cholest-5- en-3-beta-ol	*B. orientale* L.	Whole plant	GC	[13]
66.	*β*-Sitosterol (Stigmast-5-en-3-ol)	*B. penna-marina* (Maxon & C.V.Morton) Kuhn, *B. arcuatum* Remy, *B. mochaenum* G.Kunkel, *B. orientale* L., *B. asperum* (Klotzsch) J.W.Sturm, *B. blechnoides* (Lag.) C.Chr., *B. hastatum* Kaulf, *B. microphyllum* (Goldm.) C.V.Morton, *B. chilense* (Kaulf.) Mett., *B. magellanicum* (Desv.) Mett., and *B. corralense* Espinosa.	Whole plant	GC, GC-MS	[13,18]
67.	Stigmasterol (24- α-cholest-5-22-Dien-3-β-ol)	*B. orientale* L.	Whole plant	GC	[13]
68.	Campesterol (ergost-5-en-3-ol)	*B. occidentale* L., *B. binervatum* (Poir.) C.V.Morton & Lellinger, and *B. brasiliense* Desv.	Fronds	GC-MS	[12]
69.	22-Dehydrocampesterol	*B. orientale* L.	Whole plant	GC	[13]
	**Fatty acids**				
70.	Palmitic acid (Hexadecanoic acid)	*B. occidentale* L., *B. binervatum* (Poir.) C.V.Morton & Lellinger, and *B. brasiliense* Desv.	Fronds	GC-MS	[12]
71.	Methyl palmitate (Hexadecanoic acid, methyl ester)	*B. occidentale* L., *B. binervatum* (Poir.) C.V.Morton & Lellinger, and *B. brasiliense* Desv.	Fronds	GC-MS	[12]
72.	Linolenic acid (9,12,15-octadecatrienoic acid)	*B. occidentale* L., *B. binervatum* (Poir.) C.V.Morton & Lellinger, and *B. brasiliense* Desv.	Fronds	GC-MS	[12]
73.	Linoleic acid (9,12-octadecadienoic acid	*B. occidentale* L., *B. binervatum* (Poir.) C.V.Morton & Lellinger, and *B. brasiliense* Desv.	Fronds	GC-MS	[12]
74.	Oleic acid (9-octadecenoic acid).	*B. occidentale* L., *B. binervatum* (Poir.) C.V.Morton & Lellinger, and *B. brasiliense* Desv.	Fronds	GC-MS	[12]
75.	Methyl linoleate (9,12-octadecadienoic acid, methyl ester)	*B. occidentale* L., *B. binervatum* (Poir.) C.V.Morton & Lellinger, and *B. brasiliense* Desv.	Fronds	GC-MS	[12]
76.	Methyl linolenate (9,12,15-octadecatrienoic acid, methyl ester)	*B. occidentale* L., *B. binervatum* (Poir.) C.V.Morton & Lellinger, and *B. brasiliense* Desv.	Fronds	GC-MS	[12]
77.	Hexanedioic acid, bis (2-ethylhexyl) ester	*B. occidentale* L., *B. binervatum* (Poir.) C.V.Morton & Lellinger, and *B. brasiliense* Desv.	Fronds	GC-MS	[12]
78.	Tetradecanoic acid 2,3-diacetoxy-propyl ester	*B. occidentale* L., *B. binervatum* (Poir.) C.V.Morton & Lellinger, and *B. brasiliense* Desv.	Fronds	GC-MS	[12]
79.	1,2,3-Propanetricarboxylic acid 2-hydroxy-, triethyl ester	*B. orientale* L.	Whole plant	GC-MS	[23]
80.	Hexanedioic acid, mono (2-ethylhexyl) ester	*B. orientale* L.	Whole plant	GC-MS	[23]
81.	Nonanoic acid	*B. spicant* (L.) Roth	Aerial parts	GC-MS	[11]
	**Alcohols**				
82.	1-Octen-3-ol	*B. spicant* (L.) Roth	Aerial parts	GC-MS	[11]
83.	3-Octanol	*B. spicant* (L.) Roth	Aerial parts	GC-MS	[11]
84.	(*E*)-2-Octenol	*B. spicant* (L.) Roth	Aerial parts	GC-MS	[11]
85.	3,7-Dimethyloctan-3-ol	*B. spicant* (L.) Roth	Aerial parts	GC-MS	[11]
	**Aldehydes**				
86.	(*E*)-2-Heptenal	*B. spicant* (L.) Roth	Aerial parts	GC-MS	[11]
87.	2-Phenylethanal	*B. spicant* (L.) Roth	Aerial parts	GC-MS	[11]
88.	Benzaldehyde	*B. spicant* (L.) Roth	Aerial parts	GC-MS	[11]
	**Carotenoids**				
89.	Epoxy-α-ionone	*B. spicant* (L.) Roth	Aerial parts	GC-MS	[11]
90.	4-Hydroxyepoxy-β-ionol	*B. spicant* (L.) Roth	Aerial parts	GC-MS	[11]
	**Heterocyclic**				
91.	3-benzoyl-4-methyl-6-ethyl-2(1*H*)-Pyridone	*B. orientale* L.	Whole plant	GC-MS	[23]

### 3.5. Other Compounds

**Figure 10:**
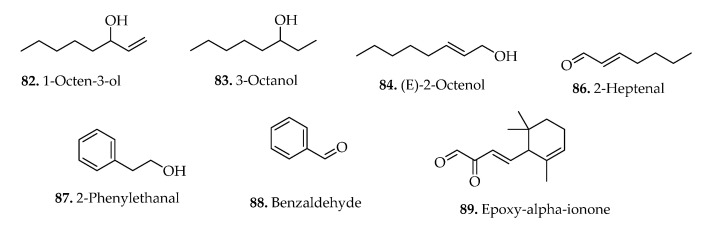
Chemical structures of alcohols, aldehydes and carotenoids from *Blechnum* species.

*Blechnum* species are significant sources of other phytochemicals including alcohols (**82**–**85**), aldehydes (**86**–**88**), carotenoids (**89**–**90**), and heterocyclic (**91**) compounds. The chemical structures of some of the isolated compounds are illustrated in Figure 10 below. The phytochemical investigation of several fern species by Fons et al. [11], using the diethyl ether extracts, resulted in detection of compounds (**86**–**90**) from the aerial parts of *B. spicant* (Table 2). In addition, a heterocyclic compound (**91**) was isolated from the whole plant of *B. orientale* [23].

## 4. Pharmacological Activity

Beneficial medicinal plants, as demonstrated by the presence of specialized metabolites, are ideal resources for revealing novel discoveries for therapeutic advances and breakthroughs. Medicinal plants serve as alternatives to modern medicines in most of the world’s populations [20]. Some *Blechnum* plants are significant sources of traditional remedies for different illnesses (Table 1), which have recently motivated the exponential increase in pharmacological research. Crude extracts and several specialized products isolated from this genus exhibit a wide range of biological activities, including the antioxidant, antimicrobial, anti-inflammatory, anticancer, wound healing, insecticidal, and antitrematocidal activities described in this section.

### 4.1. Antioxidant Activity

Antioxidants are chemical compounds that play critical roles in preventing or delaying lipid peroxidation and scavenging free radicals, which are major causes of diseases in humans and other animals [65]. The ability of medicinal plants to scavenge 2,2-diphenyl-1-picrylhydrazyl (DPPH) free radicals has been reported in the Blechnaceae family [66,67,68]. Several studies evaluated the antioxidant properties of various extracts obtained from the *Blechnum* plants. The fractions at 2–1000 µg/mL prepared from the leaves of *B. orientale* showed significant scavenging effects, comparable to the positive controls. The ethyl acetate fraction (EAf) displayed the strongest activity, equivalent to Trolox-C (IC_50_ 8.6 µg/mL) and greater than butylated hydroxytoluene (BHT) (IC_50_ 17.2 µg/mL) and tocopherol (IC_50_ 12.0 µg/mL) controls [2].

Similarly, the biological properties of hexane (HX) and dichloromethane (DCM) fractions obtained from the fronds of *B. binervatum*, *B. brasiliense,* and *B. occidentale* was tested at the concentrations of 1-500 µg/mL. Both extracts showed potent activities (<180 µg/mL), with DCM (IC_50_ 15.85 ± 1.79 µg/mL) and HX (IC_50_ 22.02 ± 1.09 µg/mL) fractions prepared from *B. brasiliense* exhibiting the highest scavenging capacity, uncomparable with the chlorogenic (IC_50_ 45.6 ± 1.52 µM) and caffeic (IC_50_ 63.3 ± 1.43) µM acid controls. Furthermore, the extracts displayed a remarkable inhibitory effect against lipoperoxidation in the homogenates prepared from rat brain (IC_50_ 56–95 µg/mL), with HX (IC_50_ 56.19 ± 1.08 µg/mL) and DCM (IC_50_ 68.48 ± 1.24 µg/mL) fractions from *B. brasiliense* exhibiting strongest activities with no harmful effects. Correspondingly, the DCM extracts showed strong inhibitory activity (IC_50_ 31.83 µg/mL) against monoamine oxidase [12].

Andrade et al. [44] investigated the in vitro antioxidant of various extracts (1–500 μg/mL) obtained from *B. binervatum*, *B. brasiliense*, and *B. occidentale* on neurodegenerative-related multi-targets. The extracts showed high scavenging abilities (IC_50_ 112.3 ± 2.61–311.1 ± 1.66 μg/mL) with *B. brasiliense* (IC_50_ 112.3 ± 2.61 μg/mL) exhibiting the strongest activity, while low activities (IC_50_ < 500 μg/mL) were revealed in nitric oxide (NO) and cholinesterase inhibitions assays. Additionally, the screened metabolites; chlorogenic (IC_50_ 37.5 ± 1.25 μM) and caffeic (55.9 ± 1.22 μM) acids demonstrated significant antioxidant activities. The cytotoxic effects were absent in both polymorphonuclear cells (PMN) and human stem cells as indicated by MTT assay. In another study, the extracts obtained from the leaves of *B. chilense* showed notable DPPH scavenging activity compared to the α-tocopherol control [39]. Tyrosinase activities of *B. orientale* extracts was moderately correlated with the antioxidant properties, supported weakly by dopachrome test [69].

Finally, the variously eluted fractions prepared from the concentrated extracts (5 g) of the whole plant of *B. orientale* using 820, 550, and 665 milligrams (mg) of HX, carbon tetrachloride, and chloroform, respectively. The elutes indicated significant antioxidant activities, with fraction VI displaying the strongest activities (64.4%) when compared to BHT (98.3%) standard. The phenolic compounds quercetin-7′,3′,4′-trimethoxy (**12**), and diterpene phytol (**42**), were associated with the exhibited antioxidant activity of *B. orientale* [23].

### 4.2. Antimicrobial Activity

A rigorous search for new antibiotics has been necessitated by the prompt development of drug-resistant strains of pathogens. Medicinal plants resources are potential candidates as antimicrobial agents, prompting extensive screenings to test their antimicrobial efficacy. In the present review, *B. orientale* was found to be an outstanding source of natural antimicrobial agents, and various investigations are discussed below.

#### 4.2.1. Antibacterial Activity

The antibacterial activities of *B. orientale* validates the traditional medicinal assertion as remedies to various infectious bacterial ailments such as ulcers, skin diseases, boils, stomach discomfort, wounds, blisters, sores, and urinary bladder complaints (Table 1). Lai et al. [2] assessed the antibacterial potential of several extracts, including crude, butanol, water, and ethyl acetate extracted from the leaves of *B. orientale*, using the broth microdilution and disc diffusion assays against 10 (Gram-positive and Gram-negative) bacterias. The tested fractions exhibited significant activities (MIC 62.5–125 µg/mL; MBC 62.5–125 µg/mL) towards *Micrococcus luteus*, *Bacillus cereus*, methicillin-susceptible *Staphylococcus aureus* (MSSA), methicillin-resistant *S. aureus* (MRSA), and *S. epidermidis.* The activities of water and butanol fractions towards *B. cereus* and *S. aureus* were comparable to other related investigations [70] and stronger (MIC 62.5 µg/mL) than the values reported by Grierson & Afolayan, [71] (MIC 500–5000 µg/mL) and Kaur & Arora, [72] (MIC 20–80 mg/mL). However, no activity was exhibited towards the Gram-negative bacterias.

Additionally, the acetone extract obtained from the leaves of *B. orientale* was tested against *S. aureus*, *Bacillus subtilis*, *Klebsiella pneumonia*, *Salmonella typhi*, *Streptococcus pyogenes*, *Proteus vulgaris*, *Pseudomonas* sp., and *Serratia* sp. at a dosage of 1 mg/disc [28]. Remarkable antibacterial activity was revealed by the maximum inhibition of *P. vulgaris* (MIC 0.025 mg/mL), while minimum zones were exhibited against *B. subtilis* and *S. aureus*. In another investigation, the leaves extract of *B. orientale* was tested against eight strains of bacterias encompassing *M. luteus*, *B. cereus*, *S. aureus* (Gram-positive), *Escherichia coli*, *Pseudomonas aeruginosa*, *Salmonella choleraesuis*, *Enterobacter aerogenes*, and *Klebsiella pneumonia* (Gram-negative), with methanol and streptomycin (10 mg) used as positive and negative controls, respectively. The three bacteria; *M. luteus* (48% inhibition, MID 500 mg/disc), *B. cereus* (57% inhibition, MID 500 mg/disc), and *S. aureus* (63% inhibition, MID 250 mg/disc), exhibited significant activities, which were comparable to streptomycin, while no inhibitions were reported for Gram-negative bacteria [72]. Insensitivity of the *Blechnum* extracts against the latter indicate the higher resistance of Gram-negative bacteria towards plant extracts compared to Gram-positive strains, consistent with other studies [73,74].

Finally, bacterial pathogens including *S. aureus, B. subtilis, S. typhi, M. luteus, P. aeruginosa* and *E. coli* were clinically isolated in an in-vitro antimicrobial efficacy trial using *B. orientale* extracts (pet-ether, chloroform, methanol, and aqueous) at 500 and 1000 µg/mL concentration. The extracts displayed remarkable inhibitions against *E. coli* at both 500 µg/mL (16.13 ± 0.09–20.2 ± 0.17 mm) and 1000 µg/mL (21.7 ± 0.16–24.0 ± 0.18 mm) concentrations, while *P. aeruginosa* showed significant inhibitions (11.0 ± 0.06–14.13 ± 0.09 mm) at 500 µg/mL. However, no inhibitions were exhibited by *S. typhi* and the rest of the Gram-positive bacterias. Both extracts were sensitive against Gram-negative bacteria, with strong sensitivity at 250 µg/mL observed in chloroform and aqueous extracts against *E. coli*. In addition, the maximum inhibitory concentration of both extracts against *P. aeruginosa* was exhibited at 250 µg/mL [19]. Thus, *Blechnum* plants are potential antibacterial agent in development of alternative phytomedicines, and this justifies the popular traditional medicinal uses in the treatment of bacterial-related infections.

#### 4.2.2. Antifungal Activity

In *Blechnum* genus, the extracts obtained from *B. orientale* were studied for antifungal activity. Deepa et al. [19] obtained various extracts prepared from the fronds at the concentration of 500 and 1000 µg/mL against *Candida albicans* and *Aspergillus flavus.* The results showed high antifungal activity against *C. albicans* at the concentration of 1000 µg/mL with 11.0 ± 0.09–12.13 ± 0.09 mm zones of inhibition compared to the standard fluconazole- 25.12 mm, while no inhibition activity was indicated towards *A. flavus*.

### 4.3. Anti-Inflammatory Activity

Inflammation might occur in the absence of illness (sterile inflammation) or as a self-defence mechanism against pathogens when the body responds to tissue injury [75,76,77]. The inflammation-related illnesses are commonly treated with steroidal or non-steroidal chemical medicines in pharmaceuticals [78]. However, these drugs have been shown to potentially induce toxicity and diverse side effects [79,80,81]. Thus, exploring new analgesic and anti-inflammatory agents with fewer negative effects is of great concern.

The various tests such as writhing, formalin, tail flick, paw oedema, and leukocyte migration were conducted to evaluate the anti-inflammatory and antinociceptive activities of the extracts derived from *B. occidentale* [3]. The mice were intraperitoneally (IP) and orally administered with the prepared extracts at the concentration ranges of 0.01–100 mg/kg and 100–400 mg/kg, respectively. The orally administered extracts dose-dependently induced writhing and showed significant antinociceptive effects. Additionally, the paw oedema induced by carrageenan and neutrophil migration was potentially reduced on intraperitoneal treatment with no cytotoxicity (*p* < 0.01).

In another study, Fasolo et al. [82] used the adult zebrafish to investigate the anti-inflammatory properties of rosmarinic acid, a bioactive molecule derived from the fronds of *B. brasiliense* by lipopolysaccharide induction model. In this trial, the intraperitoneal (i.p.) injection of lipopolysaccharides (LPS) was administered to induce a neuroinflammation cascade. The results indicated the promotion of TNF-α and IL-1β in zebrafish brain content within 24 h. Co-treatment with rosmarinic acid at the concentration of 2.5–7.5 mg/kg, and further LPS administration, showed the capability of the latter compound to reduce TNF-α and IL-1β levels. The compound exhibited a dose-dependent anti-inflammatory effects, with the highest concentration (7.5 mg/kg), greatly decreasing the TNF-α levels (*p* = 0.0026) and IL-1β levels (*p* = 0.0053) 6 h after the induction of LPS, while they were reduced in lower doses and somewhat prevented their augmentation. The investigated properties validate the traditional use of *Blechnum* species in treating various ailments, including inflammations, liver, urinary, and pulmonary infections.

### 4.4. Anticancer Activity

Cancer is among the leading cause of mortality today, and is characterized by uncontrolled growing and spreading of abnormal cells [83]. The burden of cancer incidence and deaths are globally accelerating with about 2.3 million new cases of female breast cancer reported recently to be among the most commonly diagnosed type of cancer infection [84], which results in nearly 40,000 deaths annually. Thus, various medicinal plants in the Blechnaceae family, such as the *Blechnum* plants, were tested for cancer treatment to replace modern medicines. The in vitro cytotoxic potential of different extracts ranging from 0.1 to 100 μg/mL concentration were prepared from the leaves, stems, and roots of *B. orientale* and investigated against the human breast cancer cell line (MCF-7wt.). Cells with oestrogen receptors (ER+) were models for assessing the events linked to chemotherapic responses of MCF-7wt. The cell viability of the roots, leaves, and stems extracts were 31.47%, 81.44%, 89.05% respectively. The extracts obtained from roots displayed cytotoxicity values of IC_50_ value of 32.07 ± 7.85 μg mL^−1^, with 19.1% cell mortality, and thus can be a vital source of cancer treatment [20].

Furthermore, Lai et al. [2] tested the various extracts obtained from *B. orientale* against four cancer cell lines. The butanol (Butf) (IC_50_ 27.5 µg/mL), water (Watf) (IC_50_ 33.4 µg/mL), and ethyl acetate (EAf) (IC_50_ 42.8 µg/mL) fractions revealed remarkable cytotoxic activities against the colonic adenocarcinoma cell (HT-29). However, the obtained values were weaker than *curcumin* (IC_50_ 5.4 µg/mL). In addition, notable activities were displayed by Butf fractions (IC_50_ 72.7 µg/mL) towards the human colonic carcinoma cell (HCT-116), unlike the human breast adenocarcinoma (MCF-7), human leukemia (K562), as well as liver Chang cells which demonstrated negligible cytotoxic potential (IC_50_ > 100 µg/mL).

### 4.5. Wound Healing Activity

Wounds are caused by physical, thermal, chemical, or microbial damage to living tissues, disrupting their normal functioning [85]. The wound-healing process is currently facing clinical challenges due to the observed inconsistencies at various stages [86]. The absence of effective modern medicines has instigated intense searches for alternative novel natural medicines. Many medicinal plants were scientifically proven as sources of therapeutics for wounds [87], consistent with the holistic approach by traditional systems toward curing various wound-related illnesses. However, only a single species in the *Blechnum* genus has been assessed for its wound-healing properties. Since antiquity, *B. orientale* has long been reported as a vital source of traditional remedies against wound-related complaints [31,32]. Pharmacogical studies support the traditional therapeutic assertion against skin-related ailments.

Lai et al. [22] used an in vivo assay technique to investigate the effects of water extracts obtained from the leaves of *B. orientale* on Sprague-Dawley rats wound healing. In this study, the animals were segregated into four groups, each consisting of six specimens, and were intraperitoneally administered with either 100 mg/kg of ketamine or 10 mg/kg xylazine in order to anesthetize them, followed by the subcutaneous injection of 5 mg/kg carprofen as an analgesia. The treatment groups were segregated into 1% and 2% water extracts and monitored daily. Controls were applied with the dosage of 0.20 g/wound once a day until healing/14 days. The size of wounds and the mean duration of epithelisation were significantly reduced in both cases. The 2% extract exhibited outstanding activities with wound contraction of 128.7 ± 13.4–0.8 ± 0.9 mm^2^ within 14 days, compared to 1% extract (139.7 ± 15.4–8.8 ± 0.9 mm^2^), which indicated interesting dose-dependent wound healing properties (*p* < 0.001) compared to both negative (163.7 ± 10.4–25.5 ± 6.4 mm^2^) and positive (133.7 ± 12.0–5.0 ± 0.9 mm^2^) controls dressed in the base cream and 10% povidone-iodine, respectively. Additionally, the collagen synthesis was higher in the group treated with 2% water extract compared to the vehicle group and 1% extract. More investigations are warranted to strongly authenticate the medicinal uses of *Blechnum* species.

### 4.6. Insecticidal Activity

Novel environmentally safe natural products from plants have been extensively exploited in search of alternative agents for synthetic pesticides aimed to effectively control insects [88,89]. Numerous medicinal plants were revealed to have significant insecticidal properties in the Blechnaceae family [18,90]. Monsalve et al. [26] used the ethyl acetate (EtOAc) and n-hexane (HX) fractions obtained from *B. chilense* to investigate it’s insecticidal potential. *Drosophila melanogaster* was treated with the prepared extracts at 200 to 800 ppm concentration ranges, while distilled water was used as a control. The extracts concentration-dependently affected development of the studied insects and induced larval mortality by 66.7% (EtOAc) and 63.3% (HX). At lower concentrations, the fractions showed either less mortality or less harmful effects. The isolated compound (ponasterone) was linked to the insecticidal activity of *B. chilense.* This metabolite showed a high affinity for ecdysone receptors found in Kc cells of *D. melanogaster* [60]. In addition, the prepared EtOAc extract concentration at 500 µg/mL obtained from the leaves of *B. chilense* showed toxicity (insecticidal efficacy) against *Galleria mellonella* larvae and induced premature pupal stage, exhibiting regulatory properties towards the development of larvae. The phytoecdysteroides compounds were potential constituents associated with the insecticidal property of *Blechnum* species [21]. Ecdysteroids hormones found in insects play a novel role in their metamorphosis. Since phytoecdysteroids metabolites have structural similarities with insect ecdysteroids hormones, plants containing this compounds act as remarkable anti-feedant agents with deterrent activities, and may instigate pathophysiological effects and organism death [91,92,93].

### 4.7. Antitrematocidal Activity

Anthelmintic resistance has become a global challenge, especially among farmers due to the parasitism which develops when synthetic anthelmintic drugs are frequently used. Alternatively, natural antiparasitic plant-derived products have been reported to effectively overcome resistance [5,94,95]. Pteridophytes have been demonstrated to be potential sources of trematodicidal drugs. Selected medicinal ferns, including *B. orientale,* were investigated for their in vitro antitrematodal activities [5]. The prepared eluates at 1 to 5 mg/mL concentration were examined against *Gastrothylax crumenifer* by incubating the test group with test extracts for one hour while recurrently observing their mobility, paralysis, and death. In addition, oxyclozanide (0.25 g/25 mL) was used as a standard control while a Hedon-Fleig (HF) salt solution was used as a negative control. The extracts showed the evidence of concentration-dependent increase in trematodicidal activities, and all amphistomes were killed at 5 mg/mL, similar to oxyclozanide, at the first ten minutes of incubation. The absence of mortality was reported for the entire incubation period at 1 mg/mL, while the concentration between 2–4 mg/mL caused partial mortality.

Devi et al. [23] performed an in vitro bioassay to assess the trematocidal potency of the extracts prepared from the whole plant of *B. orientale* against *G*. *crumenifer*. The test groups (25 amphistomes) were incubated with 25 mL of the extract fractions ranging from 1 to 5 mg concentration. In contrast, 0.25 g/25 mL of oxyclozanide was used as a control and visually observed at regular intervals for their mobility. Qualitative analysis indicated that the ethanol extracts concentration-dependently exhibited remarkable trematocidal activities against *G. crumenifer*. The most potent effects observed by duration taken to kill the trematodes were displayed at 5 mg concentration and were not comparable to oxyclozanide control. The screened phytochemical compounds, including quercetin-7′,3′,4′-trimethoxy (compound **12**), and phytol isomer (**42**), were associated with the antitrematocidal efficacy of *B. orientale*. These specialized metabolites targeted the tegumental regions, which caused varied changes in enzymes at predilection sites. The shifts in tegumental enzymes restricted the metabolic action of trematodes resulting in the death of worms. However, more in vitro and in vivo investigations are essential to authenticate *Blechnum* species as vital sources of novel anthelmintic plant-derived drugs.

### 4.8. Other Activities

In addition to the above-mentioned biological activities, *B. orientale* has been shown to have significant inhibitory activity (EC_50_ = 65.78 µg/mL) against α-glucosidase when compared to the myricetin control. As a result, this plant could be an interesting source of natural antidiabetic agents [96].

**Figure 11:**
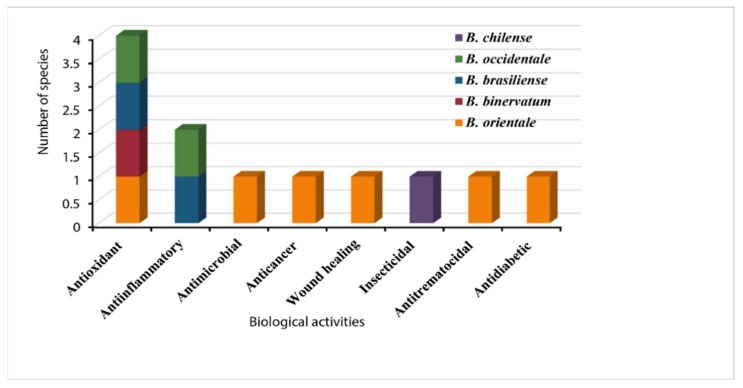
Types of *Blechnum* species with biological properties.

In the *Blechnum* genus, several species including *B. orientale, B. binervatum, B. brasiliense,* and *B. chilense* were reported to have pharmacological relevance (Figure 11). *Blechnum orientale* was widely studied, followed by *B. occidentale* and *B. brasiliense*, respectively, while *B. chilense* and *B. binervatum* were least studied. The reported biological properties including antioxidant, antimicrobial, anti-inflammatory and antinociceptive, wound healing, anticancer, and antitrematocidal activities concern different ailments and support the traditional assertion as treatments for influenza, typhoid, pulmonary infections, cancer, skin illnesses, inflammation, and intestinal wounds.

## 5. Conclusions

In the present review, we compiled information on the chemical constituents and biological activities of the *Blechnum* species. This genus encompasses several medicinally significant plants with numerous specialized metabolites such as alcohols, aldehydes, carotenoids, phenolic compounds, fatty acids, sterols, and terpenoids. A wide range of pharmacological effects such as antioxidant, antimicrobial, anti-inflammatory, anticancer, wound healing, insecticidal, and antitrematocidal properties were exhibited by the plant extracts and isolated metabolites. Several terpenoids and flavonoids metabolites were associated with antioxidant, antitrematocidal, and insecticidal activities. However, the pharmacological activities of most specialized metabolites listed in this review have yet to be investigated.

Although the genus has reported interesting medicinal uses, there are still several gaps that limit understanding of the scientific implications of several medicinally used *Blechnum* species. Foremost, only 20 of the 236 recognized species in the genus *Blechnum* have been evaluated for phytochemistry and bioactivities, while numerous other species are still unexplored. Secondly, most pharmacological analyses centered on crude extracts, with no emphasis on the specific metabolites associated with biological activities. Thirdly, in vitro models were mainly explored in assessing the pharmacological properties, with only a few studies employing the in vivo systems. In addition, the observed bioactivities were mainly random screenings with no link between traditional uses and pharmacological activities. Lastly, only a single report described the underlying modes of action of bioactive metabolites with pharmacological effects. These afore-mentioned aspects associated with the medicinal uses of the *Blechnum* species necessitate further investigations to warrant their pharmaceutical applications.

Importantly, the present review demonstrates the scientific basis for some of the medicinal uses of a variety of *Blechnum* species and supports their traditional therapeutic uses as remedies for various illnesses, validated by the majority of the pharmacological properties. Further investigations are required to extensively screen the specialized metabolites and investigate their biological activities to bring new fascinating findings and expound on the scientific basis of *Blechnum* for safe clinical application.

## Data Availability

Not applicable.
